# A Novel Successful Case of Nasal and Sinus Yolk Sac Tumor With SMARCB1 (INI-1) Deficiency: A Case Report

**DOI:** 10.7759/cureus.31320

**Published:** 2022-11-10

**Authors:** Tianyu He, Zhiyu Wang, Hongbo Su, Sihan Li, Zheng He

**Affiliations:** 1 Radiation Oncology, The First Hospital of China Medical University, Shenyang, CHN; 2 Pathology, The First Hospital and College of Basic Medical Sciences of China Medical University, Shenyang, CHN

**Keywords:** case report, chemo-radiotherapy, smarcb1-deficient carcinoma, nasal and sinus, yolk sac tumor

## Abstract

Yolk sac tumors (YSTs) of the head and neck region account for only 1% of all malignant tumors of germ cell origin. YSTs with SMARCB1 deficiency are very aggressive. Only one nasal and sinus YST with SMARCB1-deficient carcinoma (SDC) was reported with follow-up information but the patient died 20 months after diagnosis. We report a successful case treated by surgery combined with radiotherapy and limited cycles of chemotherapy, achieving a good prognosis. A 55-year-old male was seen with a three-month history of right nasal congestion, right nasal hemorrhage and hyposmia. The tumor widely invaded multiple regions such as the sphenoid, ethmoid sinus, orbital medial wall, choana, right maxillary sinus, and right pterygopalatine fossa. After endoscopic surgery, he was diagnosed as SDC with pure YST differentiation. The patient underwent endoscopic surgery, combined with radiotherapy as well as three cycles of chemotherapy with etoposide and cisplatin (EP regimen) and finally achieved over one year of disease-free survival. YST with SDC in the nasal and sinus regions is very rare and hard to treat. We highlight the value of combined treatment options including surgery, radiotherapy and limited cycles of chemotherapy to achieve a good prognosis.

## Introduction

Yolk sac tumors (YSTs) are malignant neoplasms that originate from germ cells. Only 20% of cases are encountered at extragonadal sites. Furthermore, malignant germ cell tumors of the head and neck region account for only 1% of all malignant tumors of germ cell origin [1]. SMARCB1 (INI1-1) is a tumor suppressor gene. Tumors are more aggressive in YSTs with SMARCB1-deficient carcinoma (SDC) [2]. One patient diagnosed with nasal and sinus YST with SDC died 20 months after diagnosis [3]. Here, we report a successful case treated by surgery combined with radiotherapy and limited cycles of chemotherapy, who achieved a good prognosis.

## Case presentation

**Figure 1:**
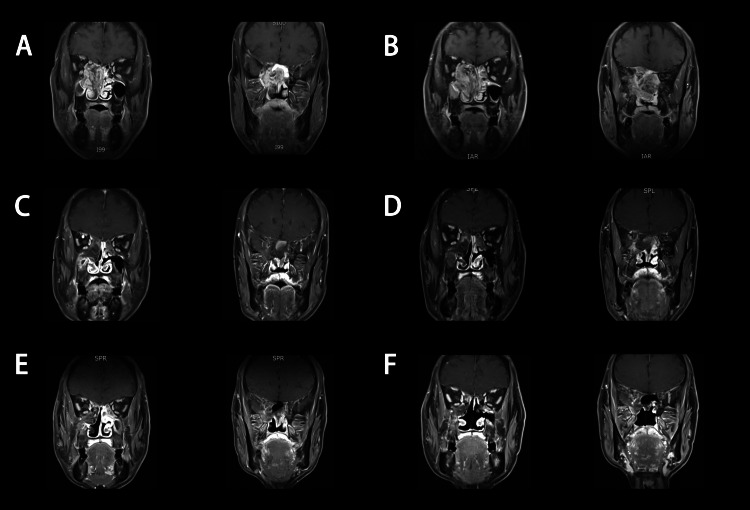
Coronal T1- weighted on the administration of contrast MR imaging At the time of diagnosis (A), 18 days after surgery (B), 1.5 months after treatment (C), three months after treatment (D), seven months after treatment (E) and 13 months after treatment (F).

A 55-year-old male was referred to our clinic. He suffered from three months of right nasal congestion, right nasal hemorrhage and hyposmia. He also presented with frequent headaches and right facial numbness. On magnetic resonance imaging (MRI), we found that the tumor invaded into the sphenoid, ethmoid sinus, orbital medial wall, choana, right maxillary sinus and right pterygopalatine fossa (Figure 1A, 2A). Significantly elevated serum a-fetoprotein (AFP) was detected as 222.3 ng/mL. The patient denied open surgery at first and an endoscopic surgery was performed. The visible tumor was removed during the operation. Li et al. published the pathological features of this case [2]. We finally diagnosed this case as SDC with pure YST differentiation.

**Figure 2:**
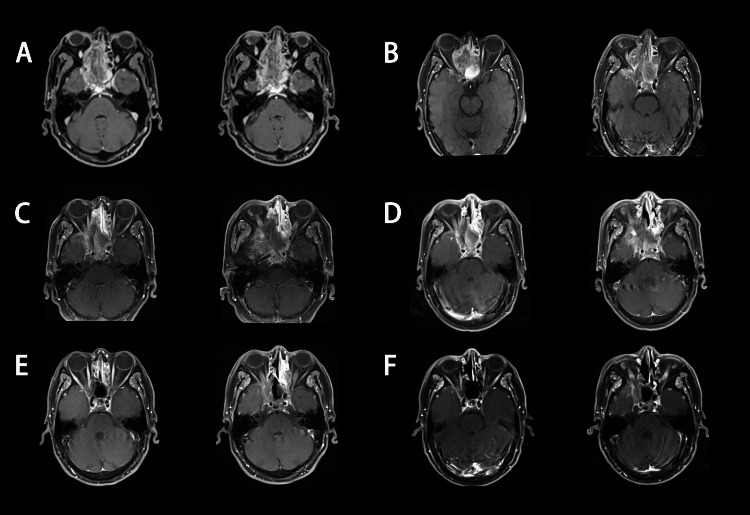
Axial T1- weighted on the administration of contrast MR imaging At the time of diagnosis (A), 18 days after surgery (B), 1.5 months after treatment (C), three months after treatment (D), seven months after treatment (E) and 13 months after treatment (F).

Eighteen days after surgical excision, an enhanced MRI was performed. Residual tumors were seen (Figures 1B, 2B). Then, we performed radiotherapy and chemotherapy. Intensity-modulated radiotherapy was carried out. Prescription doses were 60  Gy to gross tumor volume, and 50.4 Gy to clinical target volume, respectively. Due to the patient’s hepatitis B and pulmonary tuberculosis. Concurrent chemotherapy was postponed. After good control of these, he received chemotherapy with etoposide and cisplatin (EP regimen), one cycle of concurrent and two cycles of sequential every three weeks.

After treatment, the tumors gradually decreased at 1.5 months (Figure 1C, 2C), three months (Figures 1D, 2D), seven months (Figures 1E, 2E), and 13 months (Figures 1F, 2F). Meanwhile, the elevated level of AFP returned to normal and stabilized at a range of 4.28-5.23 ng/mL. During the course of treatment and follow-up, the patient had no treatment-related adverse reactions of grade III/IV. At 13 months’ follow-up, there was no evidence of recurrence and metastasis.

## Discussion

Germ cell tumors show different behaviors based on patients’ age, tumor size, location, and histological type. Kusumakumari proposed that the prognosis of extragonadal germ tumors was worse than that of gonadal tumors [4]. Roy et al. reported two cases of male children patients whose tumors were located in the head and neck region and invaded the surrounding tissue widely [5]. The management of extragonadal germ tumors varied with location heterogeneity and histological aggressiveness. YST was a kind of high-grade malignant germ cell tumor and prone to local recurrence [6].

Complete tumor excision is the first choice for patients with locally resectable tumors and will lead to good results. Two nasal and sinus YST cases showed good short-term survival after complete surgical resection combined with postoperative radiotherapy (with or without concurrent chemotherapy) [7,8]. However, usually, YST grows rapidly and invades widely. The locally advanced stage of YST makes the patient difficult to undergo radical surgery. Therefore, some patients cannot receive complete surgical resection. One nasal and sinus YST patient who only underwent chemotherapy and palliative radiotherapy showed poor response to the treatment [1]. Thus, surgery is the most important modality to treat nasal and sinus YSTs.

SMARCB1 (INI1-1) is a tumor suppressor gene. Tumors are more aggressive in YSTs with an SDC. Zamecnik et al. first reported the feature of a paranasal sinuses YST-SDC patient. She could not undergo complete surgical resection and received treatment of partial tumor resection combined with chemotherapy [9]. Unfortunately, this report showed us only the rare pathological nature of YST with SDC in adults but did not provide follow-up and survival information on the patient. Another three cases showed the same pathological feature of nasal and sinus YST-SDC and still provided no additional treatment information [10]. Last year, Hazir et al. reported a 55-year-old male with sinal YST-SDC, who underwent induction chemotherapy, complete tumor excision, and chemoradiation. Lamentedly, this patient died from chemotherapy-related leukopenia and sepsis 20 months after diagnosis [3].

To our knowledge, this is the first reported case of nasal and sinus YST-SDC successfully treated by partial resection combined with postoperative radiotherapy and limited cycles of chemotherapy, leading to over one year of disease-free survival. In our case, the patient had a wide range of tumors, which meant the tumor grew rapidly, indicating a high grade of malignancy. Endoscopic surgery was used to resect the visible tumor and identify the pathological diagnosis. The diagnosis of YST-SDC revealed that the patient may be with poor prognosis. So, we conducted a comprehensive treatment modality with radiotherapy and chemotherapy. For the reason for his complications, only three cycles of chemotherapy were conducted. Nevertheless, fortunately, this patient achieved disease-free survival over one year.

## Conclusions

Yolk sac tumors of the nasal and sinus with SMARCB1 (INI-1) deficiency are very rare in clinical practice, and usually, they are hard to treat. The success of this rare case provides us with a comprehensive treatment option including surgery, radiotherapy and limited cycles of chemotherapy.
